# Simulation of water impregnation through vertically aligned CNT forests using a molecular dynamics method

**DOI:** 10.1038/srep32262

**Published:** 2016-08-26

**Authors:** Tomohiro Tajiri, Ryosuke Matsuzaki, Yoshinobu Shimamura

**Affiliations:** 1 Tokyo University of Science, 2641 Yamazaki, Noda, Chiba 278-8510, Japan; 2 Shizuoka University, 3-5-1 Johoku, Hamamatsu, Shizuoka 432-8561, Japan

## Abstract

The flow rate of water through carbon nanotube (CNT) membranes is considerably large. Hence, CNT membranes can be used in nanofluidic applications. In this work, we performed a molecular dynamics (MD) simulation of the introduction of water into CNTs in the CNT membranes, especially in vertically aligned CNT forests. The results showed that the Knudsen number (*Kn*) increased with an increasing volume fraction of CNT (*V*_*C*_) and was greater than 10^−3^ for each *V*_*C*_. Beyond this value, the flow became a slip flow. Further, the permeability increased as *V*_*C*_ increased in the actual state calculated by the MD simulation, whereas the permeability in the no-slip state predicted by the Hagen–Poiseuille relationship decreased. Thus, a clear divergence in the permeability trend existed between the states. Finally, the flow enhancement ranged from 0.1 to 23,800, and the results show that water easily permeates as *V*_*C*_ increases.

Carbon nanotubes (CNTs) possess many excellent characteristics such as high thermal conductance, high strength, and chemical stability. Moreover, they are widely applied to electrical^1^,^2^,^3^, structural^4^,^5^,^6^, and biometric^7^,^8^,^9^ materials. Among them, vertically aligned CNT forests (VACNFs^10^) (Fig. 1) have attracted great attention because they can be produced on a large scale at low cost by chemical vapor deposition^11^, and their mesoporous structures can potentially be used in nanofluidic applications such as nanofilters, biosensors, and catalysts^12^,^13^,^14^. In the design of nanofluidic equipment based on the CNTs, it is essential to understand and control the interaction between the CNTs and the fluid^12^,^15^,^16^,^17^. Therefore, many analytical^18^,^19^,^20^,^21^ and experimental^22^,^23^,^24^,^25^,^26^ investigations on water permeation inside a CNT have been carried out. The flow rates of water through a CNT have been reported to be from one to five orders of magnitude greater than those predicted by the continuum-based no-slip Hagen–Poiseuille relationship. Furthermore, these flow rates increase as the area of the permeation region decreases. A similar trend was observed in the experimental results of Byeongho *et al.*^27^, who investigated water permeability outside a CNT in a VACNF. The similarity of the water flow inside and outside CNTs is that the flow rate and penetration coefficient increase, and the fluid flow becomes easier as the area where the fluid is impregnated becomes narrower. On the other hand, the difference is that the water flow inside a CNT is more restricted compared with that outside a CNT because the flow passes through a circular pipe. In addition, we succeed in defining the slip distance for water flow inside a CNT as well as derive the penetration coefficient formula considering the slip distance. For the water flow outside a CNT, the complex shape of the impregnation area prevents the definition of slip distance. Determining the penetration coefficient formula that considers slip is also difficult.

Although analytical and experimental flow investigations inside a CNT have been performed, no analytical investigations have been conducted outside a CNT in a VACNF. Thus, in the present study, we simulated the permeation of water outside a CNT in a VACNF and investigated the water flow. Here, we used the Gebart model^28^ as the permeability model of a porous medium, which is known for its consistency with experiments using macro porous medium and continuum numerical analyses. However, the fluid inside a minute porous medium at a nanolevel scale involves a high Knudsen number (*Kn*), and the flow turns into a slip flow^29^, which has a high velocity in the liquid/solid interface^30^. Thus, the Gebart equation cannot be applied to a fluid in a nanopore; however, this inapplicability has not been confirmed yet. Our research goal is to verify the application of the Gebart equation by comparing the permeabilities of the Gebart equation (no-slip state) (derived using a hypothesizing Hagen–Poiseuille flow) with those in an actual state that uses molecular dynamics (MD) results and to verify the flow tendency in a nanopore.

In this study, we consider a fluid water flow with a flow front; the flow is induced by a capillary force outside a CNT. Moreover, we present our results in the following aspects: (1) investigation of the Knudsen number (*Kn*), (2) verification of the application of permeability predicted by a no-slip state, and (3) investigation of the divergence between the permeability in the slip and actual states by calculating the flow enhancement in a nanopore.

## Results

### Analytical model

**Figure 2:**
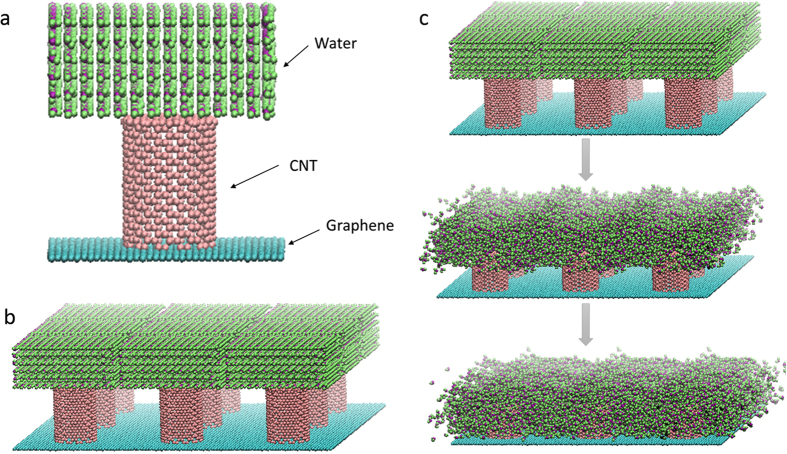
Analytical models and permeating behavior. (**a**) Analytical model of impregnation outside a CNT. (**b**) Impregnation is performed by applying the periodic boundary condition. (**c**) Behavior of permeating water outside CNTs.

The analytical model of the permeation outside the CNT is shown in Fig. 2(a). We set the CNT with a cap at the center of a graphene and arranged water molecules above the CNT. The CNT was fixed because the CNT and graphene were bonded between carbon atoms. The CNT diameter was 2.16 nm, and its length was 3.19 nm. We varied the size of the graphene to obtain *V*_*C*_ = 0.077, 0.090, 0.106, 0.188, 0.311, 0.424, 0.505, 0.611, 0.706, and 0.780. Here, we defined *V*_*C*_ as *V*_*C*_ = (area of the CNT)/(area of graphene) in the model shown in Fig. 2(a). By applying periodic boundary conditions to the analytical model shown in Fig. 2(a), we created the VACNF shown in Fig. 2(b) and performed permeation outside the CNT, as shown in Fig. 2(c). Periodic boundary conditions were applied along every boundary of the unit cell. We established a sufficiently long distance with respect to the CNT axis so that the model duplicated in each direction does not affect the water molecules in the cell. Then, we investigated the impregnation of water through capillarity and did not apply any force to the impregnation.

### Fluid permeation outside CNTs

In the evaluation formula of fluid permeation of the actual state calculated by the MD simulation, we used the following permeability in the Darcy’s law^31^ [equation (1)]:


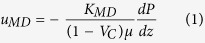


where *u*_*MD*_ is the velocity of the fluid permeation into porous media; *K*_*MD*_ is the permeability, which is calculated by the MD simulation; *μ* is the viscosity of the fluid, and *dP/*d*z* is the pressure gradient in the flow direction.

The Gebart equation^28^ [equation (2)] is widely used to verify the macroscopic-pore permeation behavior in case the permeation direction is parallel to the fibrous direction under the assumption of a no-slip flow.


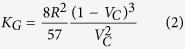


**Figure 3:**
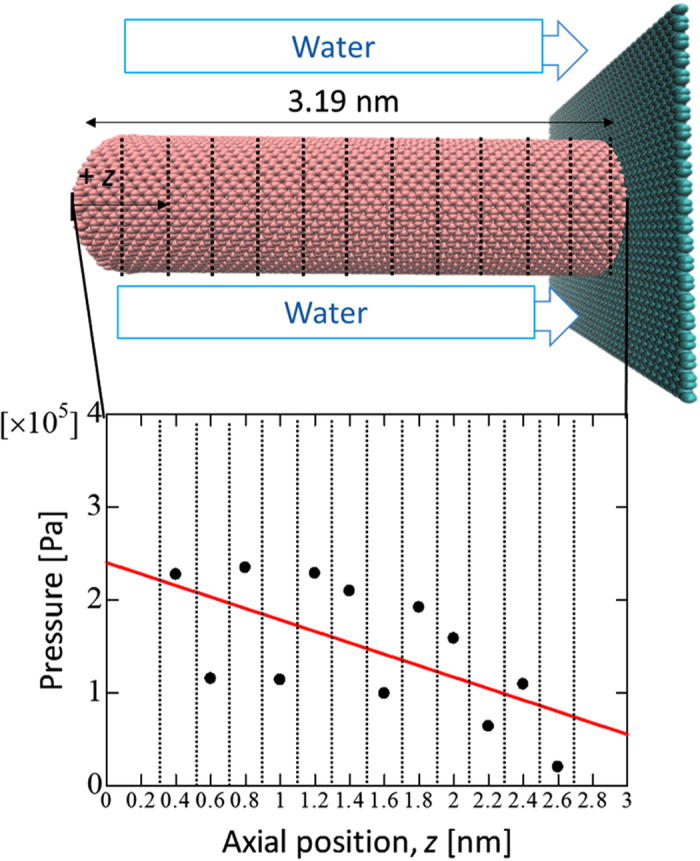
Pressure gradient. Axial pressure gradient between the CNTs at intervals of 0.2 nm. d*P*/d*z* is calculated by evaluating the pressure within several sub-volumes along the CNT axis and performing a linear regression analysis.

*K*_*G*_ is the permeability calculated by the Gebart equation, and *R* is the fiber radius. Then, we considered *K*_*MD*_ to be the actual permeability and *K*_*G*_ to be the permeability of the no-slip state. We compared the permeability obtained from equation (1) using the MD simulation with that from equation (2). Furthermore, as shown in Fig. 3, we calculated d*P*/d*z* by evaluating the pressure within several sub-volumes along the CNT axis and performing a linear regression analysis. Here, we calculated the pressure in the MD using the virial theorem [equation (3)]^32^.


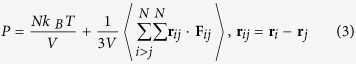


where *V* is the volume, *N* is the number of atoms, *k*_*B*_ is the Boltzmann constant, *T* is the temperature, **r**_*ij*_ is the distance between atoms *i* and *j*, and **F**_*ij*_ is the interaction force between atoms *i* and *j*.

In the case of a liquid slip at the solid/liquid boundary, the actual permeability (*K*_actual_, measured from the experiment or predicted from the MD simulation) becomes larger than the calculated value from the Hagen–Poiseuille relationship under the assumption of no-slip flow (*K*_no-slip_)^19^. This increase in *K* leads to the definition of flow enhancement (*ε*) given by equation (4)^19^.


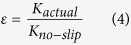


We consider *K*_*actual*_ as *K*_*MD*_ and *K*_*no-slip*_ as *K*_*G*_ in this paper.

### Results of the MD simulation and calculation

**Figure 4:**
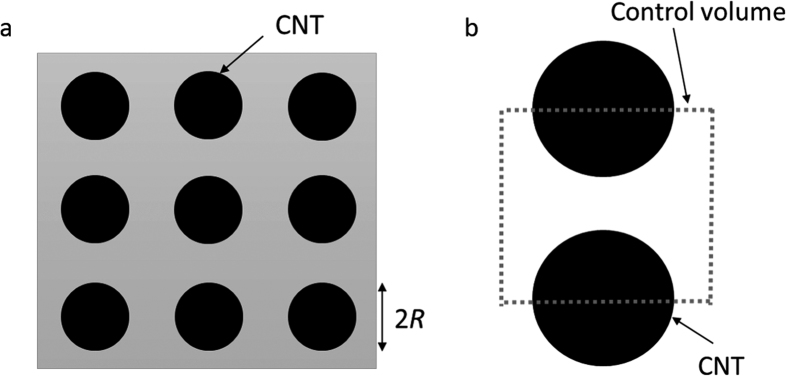
Results of the MD simulation and calculation. (**a**) Velocity (*V*_*C*_ = 0.106) of the CNT axial direction in position *r* from the CNT center, and its velocity profile is calculated in the domain from *z* = 0.5 nm to *z* = 1.0 nm. The vertical dashed line at *y* = *h* represents the CNT surface. (**b**) *V*_*c*_ versus velocity of water along the impregnation direction. Its velocity is calculated in the computational domain from *z* = 0.5 nm to *z* = 1.0 nm. *V*_*C*_ versus (**c**) *Kn*, (**d**) permeability, and (**e**) enhancement.

We show the results of the MD simulation and the calculation in Fig. 4. We make three analysis attempts for each *V*_*c*_, and the data shown in each of the figures are its average values. First, Fig. 4(a) shows the result of the mean velocity of water in the CNT axial direction at position *r* from the CNT center. The velocity profile is calculated in the domain from *z* = 0.5 nm to *z* = 1.0 nm at *V*_*C*_ = 0.106. In this figure, the velocity becomes maximum near the CNT surface and minimum near the center between the CNTs. We find that a recess-shaped meniscus is formed. The same tendency is observed for other *V*_*C*_ values. Figure 4(b) shows the result of the average permeation velocity for all the water molecules in the CNT axial direction. The figure shows that the permeation velocity does not monotonically changed as the permeation area varies, and the flow tendency across *V*_*C*_ = 0.106 varies. In Fig. 4(c) *Kn* is higher than 0.01 for *V*_*C*_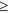 0.188, and in Fig. 4(a), velocities exist at the CNT surface. Thus, we find that the water flow becomes a slip flow. Furthermore, *Kn* increases as the permeation area becomes narrower.

Figure 4(d) shows the relationship between *V*_*C*_ and permeability (log *K*). In this graph, although the actual permeability using the MD results increases, that of the no-slip state decreases as *V*_*C*_ increases or the permeation area becomes narrower. On the other hand, at *Vc* < 0.188, the permeability of the actual state using the MD results corresponds with that of the no-slip state. Finally, Fig. 4(e) shows the relationship between *V*_*C*_ and the flow enhancement. The range of the enhancement obtained in this research is 0.1–23800. In some areas, the permeability is more than 10^3^ compared with that in the no-slip state. Furthermore, we find that the enhancement increases, and water more easily permeates.

## Discussion

First, Fig. 4(a) shows that the velocity profile formed a recess-shaped meniscus. Similarly, the experimental results of Rossi^33^, who investigated the flow front of water inside a CNT using environmental scanning electron microscopy, also showed that water formed a meniscus. In the present research, we considered the water flow with a flow front, and in this flow, the surface tension exerted a strong influence. The velocity distribution was meniscus, although this does not necessarily mean that the shape is meniscus. However, because the MD model in this study assumes ordinary temperatures, the model assumes an atmosphere rather than a vacuum in areas containing no molecules. In other words, because the liquid and vapor layers co-exist, we believe that meniscus formation can occur, and the result of the mean velocity can accurately represent the permeation behavior. Moreover, Kenneth *et al.*^34^ experimentally showed that the surface of the VACNF, which has an array of CNTs of not more than tens of micrometers, is not sufficiently hydrophobic by itself, and water droplets deposited on the surface permeate into the forest. On the other hand, in the CNT array of more than hundreds of micrometers, the top surface of the CNTs is superhydrophobic, and water does not permeate into the forest. Because we used a 3.19-nm-long CNT in the VACNF, we deduce that no contradiction occurs in the result of water permeation into the VACNF in the MD simulation.

Second, from the trend shown in Fig. 4(d), we can state that a clear divergence in the permeability tendency occurs between the slip and no-slip states, and the permeabilities show an opposite trend with *V*_*C*_. The trend in which the permeability increases and the fluid flows more easily as the permeation area becomes narrower corresponds to the experimental results of Byeongho *et al.*^27^ who investigated water permeability in a VACNF. On the other hand, the permeability of the slip state corresponds to that of the no-slip state as the permeation area becomes wider. According to the above results, in the permeation in nanopore-based CNTs, a wide region exists in *V*_*C*_ where permeability in the no-slip state cannot be applied; therefore, considering a new permeability equation with slip is necessary. Then, a wide area is necessary to prove the pressure statistics, but in the present study, we averaged over a very narrow calculation area. To clarify the effect of the variation due to the limited calculation area, we conducted an MD analysis three times under each condition. As a result, we showed that the penetration coefficient was not affected, which is the topic of this paper.

Finally, from the result of the enhancement shown in Fig. 4(e), a wide region exists in *V*_*C*_ where the permeability is more than 10^3^ compared with that in the no-slip state because the superhydrophobicity of the CNT generated almost zero friction. Moreover, weak interfacial forces exist between the CNT and water molecules, resulting in a very large enhancement. This phenomenon was believed to occur due to the superhydrophobic surface property of CNT. However, to clarify this phenomenon even further, we believe an indicator is necessary to quantitatively evaluate hydrophobism and hydrophilism. However, this index is still currently under consideration and can be studied in the future. On the other hand, we believe that extremely valuable elements are present in the current discussion that show the applicability of the Gebart formula assuming a no-slip state derived by the Navier–Stokes equations as well as the trend in liquidity outside the CNT in the model that simulates the VACNF.

## Methods

### Knudsen number

For gases, *Kn* is defined as *Kn* = *λ*/*L*_*s*_, where *λ* is the mean free path in a gas and *L*_*s*_ is the characteristic channel dimension. *Kn* is used to determine whether a flow field is a continuum^35^. In this study, the equivalent Knudsen number is calculated using lattice spacing *δ* according to ref. 36 instead of using the mean free path. This method of determining the Knudsen number is used because liquid molecules do not exhibit a mean free path. Here, the lattice spacing *δ* is defined as^37^


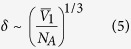


where 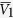 is the molar volume and *N*_*A*_ is Avogadro’s number. For liquid water, because 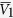 is 18 cm^3^, the lattice spacing is 0.3 nm. We used equation (6) to determine *Kn* for the permeation outside the CNT.


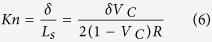


**Figure 5:**
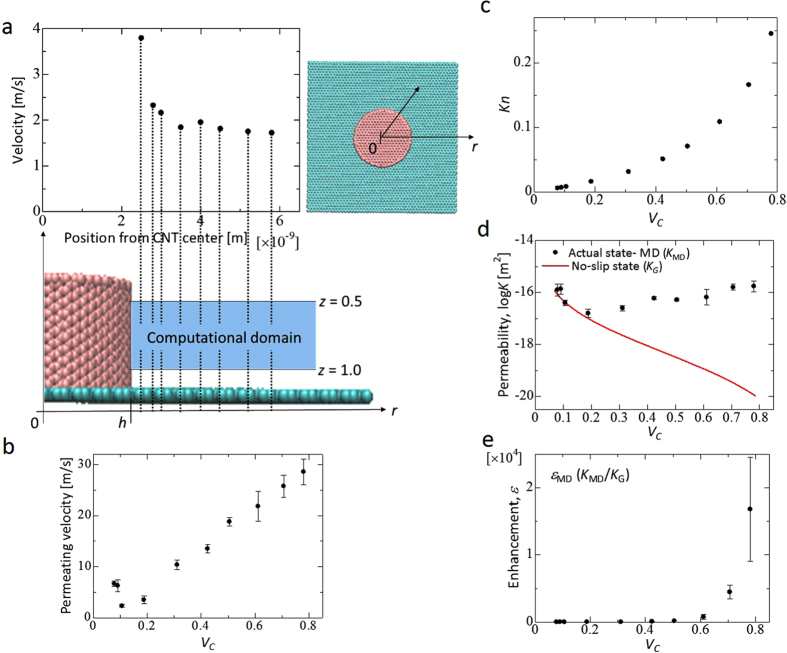
Definition of the control volume to calculate the hydraulic diameter. (**a**) Top view of the interface in a two-dimensional patterning surface with hydrophobic posts organized on a square lattice. (**b**) Definition of the control volume.

*L*_*s*_ corresponds to the hydraulic diameter^28^ that considers the control volume [Fig. 5(b)] of the model where the CNTs stand close together and straight on the substrate [Fig. 5(a)]. Here, many cases exist where the flow is approximated by reflecting the slip condition of the Navier–Stokes equations for nanoscale liquid flow^30^. The Gebart model used here utilizes the Navier–Stokes equation assuming no-slip conditions. We expect that the Gebart model will no longer be applicable once *Kn* > 0.01 38 because differences will appear under these conditions. Considering these points, we utilize *Kn* as a condition for determination.

### Molecular simulation

In the investigation, we conducted an MD simulation using the LAMMPS^39^ and permeated water outside a CNT in the VACNF. In the simulation, we used the TIP3P model^40^,^41^,^42^ for the water and AMBER96 43 for the potential function. The viscosity of water was *μ* = 0.321 mPa·s at 300 K^30^. The long-range Coulomb forces were computed using the particle–particle particle-mesh method^44^, and the mean square error was 10^−4^. The SHAKE method^45^ was used to solve the equation of motion under a constrained condition. We used the *NVT* ensemble as the statistics ensemble and controlled the system temperature by solving the Langevin equation of motion^46^ in the relaxation calculation and permeation simulation. We used the values listed in Table 1 for each parameter in the potential function and analysis conditions in the relaxation calculation. The permeation simulation conditions are listed in Table 2.

## Additional Information

**How to cite this article**: Tajiri, T. *et al.* Simulation of water impregnation through vertically aligned CNT forests using a molecular dynamics method. *Sci. Rep.* **6**, 32262; doi: 10.1038/srep32262 (2016).

## Figures and Tables

**Figure 1:**
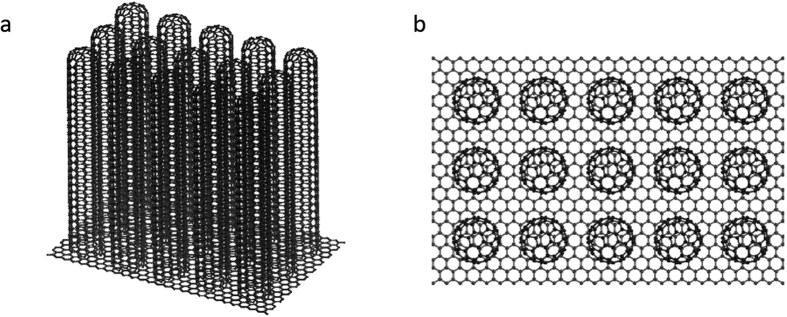
VACNFs. (**a**) General and (**b**) top views of a VACNF.

**Table 1:** Parameters of AMBER96 potential function.

Atoms(*ij*)	*ε*_*ij*_ [kcal/mol]	*σ*_*ij*_ [Å]
CC	0.086	3.407
OO	0.1521	3.151
HH	0.0	0.0
CO	0.114	3.275
CH	0.0	0.0
Bonds(*ij*)	*K*_*ij*_^*R*^ [kcal/mol/Å^2^]	*R*_*ij*_^*eq*^ [Å^2^]
OH	1000.0	1.0
Angles(*ijk*)	*K*_*ijk*_^*θ*^[kcal/mol/rad^2^]	*θ*_*ijk*_^*eq*^ [°]
HOH	1,000.0	109.47

*i*, *j*, and *k* represent an atom.

**Table 2:** Computational condition of simulation.

Relaxation	Time step [fs]	0.01
	Running time [fs]	20
	Damping constant	100.0
Simulation	Time step [fs]	1.0
	Running time [fs]	8.0 × 10^5^
	Temperature [K]	300

## References

[b1] Yamada T. *et al.* A stretchable carbon nanotube strain sensor for human-motion detection. Nature Nanotechnology 6, 296–301 (2011). 10.1038/nnano.2011.36 21441912

[b2] Izadi-Najafabadi A. *et al.* Extracting the full potential of single-walled carbon nanotubes as durable supercapacitor electrodes operable at 4 V with high power and energy density. Advanced Materials 22, E235–E241 (2010). 20564700 10.1002/adma.200904349

[b3] Chaban V. Filling carbon nanotubes with liquid acetonitrile. Chemical Physics Letters 496, 50–55 (2010).

[b4] Silvestre N., Faria B. & Canongia Lopes J. N. Compressive behavior of CNT-reinforced aluminum composites using molecular dynamics. Composites Science and Technology 90, 16–24 (2014).

[b5] Shimamura Y. *et al.* Tensile mechanical properties of carbon nanotube/epoxy composite fabricated by pultrusion of carbon nanotube spun yarn preform. Composite: Part A 62, 32–38 (2014).

[b6] Grujicic M., Sun Y. P. & Koudela K. L. The effect of covalent functionalization of carbon nanotube reinforcements on the atomic-level mechanical properties of poly-vinyl-ester-epoxy. Applied Surface Science 253, 3009–3021 (2007).

[b7] Xiao Z. *et al.* Base- and structure-dependent DNA dinucleotide-carbon nanotube interactions: Molecular dynamics simulations and thermodynamic analysis. The Journal of Physical Chemistry 115, 21546–21558 (2011).

[b8] Shi X., von dem Bussche A., Hurt R. H., Kane A. B. & Gao H. Cell entry of one-dimensional nanomaterials occurs by tip recognition and rotation. Nature Nanotechnology 6, 714–719 (2011). 10.1038/nnano.2011.151 PMC3215144 21926979

[b9] Ryman-Rasmussen J. P. *et al.* Inhaled carbon nanotubes reach the subpleural tissue in mice. Nature Nanotechnology 4, 747–751 (2009). 10.1038/nnano.2009.305 PMC2783215 19893520

[b10] Kenji H. *et al.* Water-assisted highly efficient synthesis of impurity-free single-walled carbon nanotubes. Science 306, 1362–1364 (2004). 15550668 10.1126/science.1104962

[b11] Abdalla S., Al-Marzouki F., Al-Ghamdi A. A. & Abdel-Daiem A. Different technical applications of carbon nanotubes. Nanoscale Research Letters 10, 358 (2015). 26377211 10.1186/s11671-015-1056-3 PMC4573081

[b12] Babu J. S. & Sathian S. P. The role of activation energy and reduced viscosity on the enhancement of water flow through carbon nanotubes. The Journal of Chemical Physics 134, 194509 (2011). 21599075 10.1063/1.3592532

[b13] Dresselhaus M. S., Dresselhaus G., Saito R. & Jorio A. Raman spectroscopy of carbon nanotubes. Physics Reports 409, 47–99 (2005).

[b14] Villalpando F. *et al.* Raman spectroscopy study of isolated double-walled carbon nanotubes with different metallic and semiconducting configurations. Nano Letters 8, 3870–3886 (2008). 18937518 10.1021/nl802306t

[b15] Su J., Yang K. & Huang D. Ultra-fast single-file transport of a simple liquid beyond the collective behavior zone. Physical Chemistry Chemical Physics doi: 10.1039/c5cp07253k (2016). 27460013

[b16] Joseph S. & Aluru N. R. Why Are Carbon Nanotubes Fast Transporters of Water? Nano Letters 8, 452–458 (2008). 18189436 10.1021/nl072385q

[b17] Mattia D. & Gogotsi Y. Review: static and dynamic behavior of liquids inside carbon nanotubes. Microfluid Nanofluid 5, 289–305 (2008).

[b18] Thomas J. A. & Mcgaughey A. J. H. Reassessing fast water transport through carbon nanotubes. Nano Letters 8, 2788–2793 (2008). 18665654 10.1021/nl8013617

[b19] Thomas J. A. & Mcgaughey A. J. H. Water flow in carbon nanotubes: Transition to subcontinuum transport. Physical Review Letters 102, 184502 (2009). 19518876 10.1103/PhysRevLett.102.184502

[b20] Su J. & Guo H. Effect of nanochannel dimension on the transport of water molecules. The Journal of Physical Chemistry 116, 5925–5932 (2012). 22448756 10.1021/jp211650s

[b21] Walther J. H., Ritos K., Eduardo R. C., Megaridis C. M. & Koumoutsakos P. Barriers to superfast water transport in carbon nanotube membranes. Nano Letters 13, 1910–1914 (2013). 23521014 10.1021/nl304000k

[b22] Holt J. K. *et al.* Fast mass transport through sub-2-nanometer carbon nanotubes. Science. 312, 1034–1037 (2006). 16709781 10.1126/science.1126298

[b23] Majumder M., Chopra N., Andrews R. & Hinds B. Nanoscale hydrodynamics: Enhanced flow in carbon nanotubes. Nature 438, 44 (2005). 16267546 10.1038/43844a

[b24] Majumder M., Chopra N. & Hinds B. J. Mass transport through carbon nanotube membranes in three different regimes: Ionic diffusion and gas and liquid flow. ACS Nano 5, 3867–3877 (2011). 21500837 10.1021/nn200222g

[b25] Qin X., Yuan Q., Zhao Y., Xie S. & Liu Z. Measurement of the rate of water translocation through carbon Nanotubes. Nano Letters 11, 2173–2177 (2011). 21462938 10.1021/nl200843g

[b26] Whitby M., Cagnon L., Thanou M. & Quirke N. Enhanced fluid flow through nanoscale carbon pipes. Nano Letters 8, 2632–2637 (2008). 18680352 10.1021/nl080705f

[b27] Lee B. *et al.* A carbon nanotube wall membrane for water treatment. Nature Communications 6, 7109 (2015). 10.1038/ncomms8109 25971895

[b28] Gebart B. R. Permeability of unidirectional reinforcements for RTM. Journal of Composite Materials 26, 1100–1133 (1992).

[b29] Cao B. Y., Sun J., Chen M. & Guo Z. Y. Molecular Momentum Transport at Fluid-Solid Interfaces in MEMS/NEMS: A Review. Int. J. Mol. Sci. 10, 4638–4706 (2009). 20087458 10.3390/ijms10114638 PMC2808004

[b30] Joly L. Capillary filling with giant liquid/solid: Dynamics of water uptake by carbon nanotubes. The Journal of Chemical Physics 135, 214705 (2011). 22149809 10.1063/1.3664622

[b31] Wang S. *et al.* Nanoscale infiltration behavior and through-thickness permeability of carbon nanotube buckypapers. Nanotechnology 24, 015704 (2013). 23221271 10.1088/0957-4484/24/1/015704

[b32] Thompson A. P., Plimpton S. J. & Mattson W., General formulation of pressure and stress tensor for arbitrary many-body interaction potentials under periodic boundary conditions. The Journal of Chemical Physics 131, 154107 (2009). 20568847 10.1063/1.3245303

[b33] Rossi P. M. *et al.* Environmental scanning electron microscopy study of water in carbon nanopipes. Nano Letters 4, 989–993 (2004).

[b34] Lau K. K. S. *et al.* Superhydrophobic carbon nanotube forests, Nano Letters 3, 1701–1705 (2003).

[b35] Willis D. R. Sphere drag at high Knudsen number and low Mach number. Physics of Fluids 9, 2522 (1966).

[b36] Gad-el-Hak M. MEMS: Introduction and Fundamentals, 2005, CRC Press.

[b37] Probstein R. F. Physicochemical Hydrodynamics: An Introduction, 2nd ed., John Wiley & Sons, Inc., New York (1994).

[b38] Kohl M. J., Abdel-Khalik S. I., Jeter S. M. & Sadowski D. L. An experimental investigation of microchannel flow with internal pressure measurements. International Journal of Heat and Mass Transfer 48, 1518–1533 (2005).

[b39] Wang X., Liu H., Wang J., Zhang W. & Li Z. Package heat dissipation with carbon nanotube micro heat sink. IEEE Conference Publications doi: 10.1109/ICEPT.2009.5270792, 73–76 (2009).

[b40] Wang L. Molecular dynamics—Studies of synthetic and biological macromolecules. Intech Journals (2012).

[b41] Jorgensen W. L., Chandrasekhar J., Madura J. D., Impey R. W. & Klein M. L. Comparison of simple potential functions for simulating liquid water. The Journal of Chemical Physics 79, 926–935 (1983).

[b42] González M. A. & Abascal J. L. The shear viscosity of rigid water models. The Journal of Chemical Physics 132, 096101 (2010). 20210414 10.1063/1.3330544

[b43] Cornell W. D. *et al.* A second generation force field for the simulation of proteins, nucleic acids, and organic molecules. Journal of the American Chemical Society 117, 5179–5197 (1995).

[b44] Yoshikawa K. & Fukushige T. PPPM and TreePM methods on GRAPE systems for cosmological N-body simulations, Publications of the Astronomical Society of Japan 57, 849–860 (2005).

[b45] Andersen H. C. Rattle: A “velocity” version of the shake algorithm for molecular dynamics calculations. Journal of Computational Physics 52, 24–34 (1983).

[b46] Jensen N. G. & Farago O. A simple and effective verlet-type algorithm for simulating Langevin dynamics. Molecular Physics 111, 983–991 (2013).

